# Tall tails: cryo-electron microscopy of phage tail DNA ejection conduits

**DOI:** 10.1042/BST20210799

**Published:** 2022-02-07

**Authors:** Joshua M. Hardy, Rhys A. Dunstan, Trevor Lithgow, Fasséli Coulibaly

**Affiliations:** 1 Chemical Biology Division, Walter and Eliza Hall Institute of Medical Research, Parkville, Victoria, Australia; 2 Department of Medical Biology, University of Melbourne, Parkville, Victoria, Australia; 3 Centre to Impact AMR, Infection Program, Biomedicine Discovery Institute & Department of Microbiology, Monash University, Clayton, Victoria, Australia; 4 Infection Program, Biomedicine Discovery Institute & Department of Biochemistry and Molecular Biology, Monash University, Clayton, Victoria, Australia

**Keywords:** bacteriophages, cryo-electron microscopy, protein structure, structural biology, virology

## Abstract

The majority of phages, viruses that infect prokaryotes, inject their genomic material into their host through a tubular assembly known as a tail. Despite the genomic diversity of tailed phages, only three morphological archetypes have been described: contractile tails of *Myoviridae*-like phages; short non-contractile tails of *Podoviridae*-like phages; and long and flexible non-contractile tails of *Siphoviridae*-like phages. While early cryo-electron microscopy (cryo-EM) work elucidated the organisation of the syringe-like injection mechanism of contractile tails, the intrinsic flexibility of the long non-contractile tails prevented high-resolution structural determination. In 2020, four cryo-EM structures of *Siphoviridae*-like tail tubes were solved and revealed common themes and divergences. The central tube is structurally conserved and homologous to the hexameric rings of the tail tube protein (TTP) also found in contractile tails, bacterial pyocins, and type VI secretion systems. The interior surface of the tube presents analogous motifs of negatively charged amino acids proposed to facilitate ratcheting of the DNA during genome ejection. The lack of a conformational change upon genome ejection implicates the tape measure protein in triggering genome release. A distinctive feature of *Siphoviridae*-like tails is their flexibility. This results from loose inter-ring connections that can asymmetrically stretch on one side to allow bending and flexing of the tube without breaking. The outer surface of the tube differs greatly and may be smooth or rugged due to additional Ig-like domains in TTP. Some of these variable domains may contribute to adsorption of the phage to prokaryotic and eukaryotic cell surfaces affecting tropism and virulence.

## Introduction

Bacteriophages, or phages, infect prokaryotes and are the most abundant biological entity on the planet [1]. Despite their ubiquity, only a fraction of the diversity in this ‘dark matter of the biosphere’ is catalogued, and most of that is focused on the phages infecting the Bacteria rather than the Archaea [2]. Phages have a central role in the ecology of bacterial populations, contributing significantly to selection pressure for genetic diversity and shaping of population structures [3]. Phages have been used as model systems for molecular biology and virology, and various lineages of phages have gained a growing importance in biotechnological and biomedical applications [4–6].

Phages have various morphologies with a pioneering electron microscopy survey of 5568 phages finding that an overwhelming majority had icosahedral capsids attached to tails, with the remainder being polyhedral, filamentous, or pleomorphic [7]. Yet more recent metagenomic studies suggest we are only yet seeing the tip of the iceberg for phage genomic and structural diversity [8–11]. The icosahedral symmetry of the genome-packaging head is not unexpected given its prevalence in spherical viruses found across the virosphere. However, tails are a specificity of archaeal and bacterial viruses, rare if not completely absent from viruses infecting eukaryotic organisms [12]. While the selective advantage of these appendages remains a matter of debate, it is clear that the major function of the tail is to deliver the genome into the cytoplasm of the host cell, and this may be achieved efficiently using the tube-like structure of the tail. Tails also project the receptor binding components away from the capsid, contributing to an increased radius of capture of their target [13].

**Figure 1.**
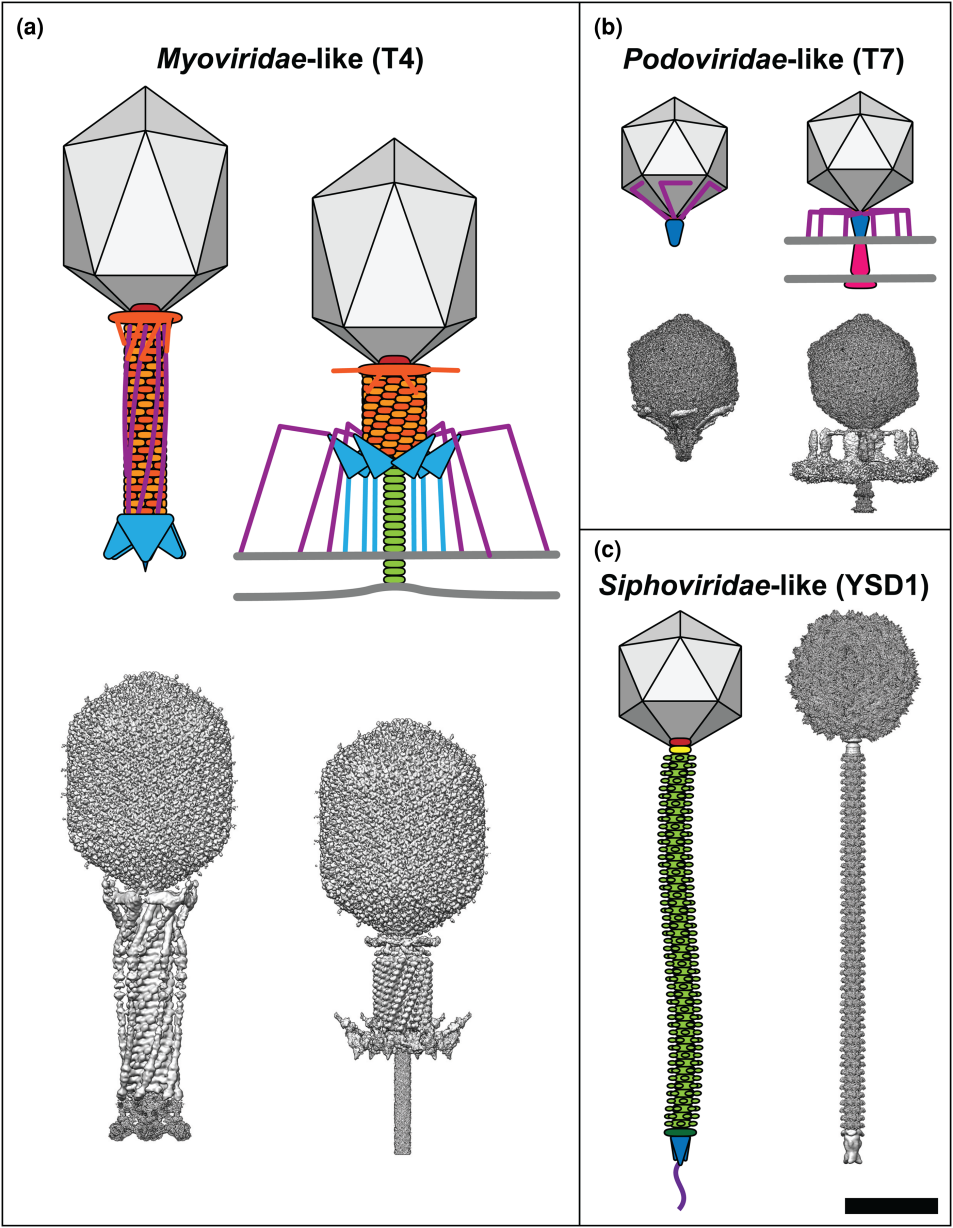
Comparison of tailed phage morphology. For each family of tailed phages a cartoon schematic is shown alongside a composite of the corresponding cryo-EM reconstructions (where available). Phages are drawn to scale and their component parts are coloured according to function: capsid, grey; portal, red; tail sheath, orange; tail terminator, yellow; tail tube, light green; distal tail protein, dark green; baseplate, cyan; tail spike, blue; tail fibres, purple; ejectosome, pink. (**a**) *Myoviridae*-like phages such as T4 have contractile tails, (**b**) *Podoviridae*-like viruses such as T7 have very short non-contractile tails and assemble an ejectosome for genome delivery, and (**c**) *Siphoviridae*-like phages such as YSD1 have long, flexible non-contractile tails. T4 maps before host attachment and genome ejection: capsid, EMD-6323 [25]; tail and sheath, EMD-1126 [26]; baseplate, EMD-3374 [27]. T4 maps after host attachment and sheath contraction: capsid, EMD-6323 [25]; sheath, EMD-5528 [28]; baseplate, EMD-3396 [27]; tail tube, EMD-8786 [29]. T7 maps before host attachment and genome ejection: capsid, EMD-30139 [30]; portal–tail complex, EMD-31319 [31]; tail fibres, EMD-31315 [31]. T7 maps after host attachment and genome ejection: capsid, EMD-30139 [30]; portal–tail complex, EMD-31321 [31]; tail fibres, EMD-31318 [31]; ejectosome, EMD-22680 [32]. YSD1 maps before host attachment and genome ejection: capsid, EMD-22182 [33]; portal/connector, unpublished data; tail, EMD-22183 [33]; spike, unpublished data. Scale bar = 50 nm. Composite maps were generated in UCSF Chimera version 1.14 [34].

Phage tails exhibit great diversity in their structures and function, features exploited in the early days of phage classification to divide tailed phages in three viral families that differ by their tail morphology: *Myoviridae*, with contractile tails; *Podoviridae*, with very short non-contractile tails; and *Siphoviridae* with long, flexible non-contractile tails (Figure 1) [14]. There are now 14 families in the Caudovirales order based on genomic analysis, but this review will refer to phages according to their structural archetype, e.g. *Myoviridae*-like (Table 1) [15].

**Table 1.** Revised taxonomy of tailed phages as established by the International Committee on Taxonomy of Viruses (ICTV) [15]

Phage archetype	Family	Example phage	Accession number
*Siphoviridae*-like	*Demerecviridae*	*Escherichia* virus T5	AY543070
	*Drexlerviridae*	*Escherichia* virus T1	AY216660
	*Siphoviridae*	*Escherichia* virus λ	J02459
*Myoviridae*-like	*Ackermannviridae*	*Escherichia* virus CBA120	JN593240
	*Chaseviridae*	*Escherichia* virus 4HA13	NC_049466
	*Herelleviridae*	*Bacillus* virus SPO1	NC_011421
	*Myoviridae*	*Escherichia* virus T4	AF158101
*Podoviridae*-like	*Autographiviridae*	*Escherichia* virus T7	NC_001604
	*Guelinviridae*	*Clostridium* virus CPS2	NC_048707
	*Podoviridae*	*Salmonella* virus P22	KR296686
	*Rountreeviridae*	*Staphylococcus* virus 66	NC_007046
	*Salasmaviridae*	*Bacillus* virus Aurora	NC_031121
	*Schitoviridae*	*Pectobacterium* virus CB1	NC_048653
	*Zobellviridae*	*Citrobacter* virus CVT22	NC_027988

The icosahedral capsids are isometric or prolate with triangulation (*T*) numbers ranging from *T* = 3 to at least *T* = 52 [14,16]. Prolate heads are elongated along one of the 5-fold axes with additional hexameric units, as seen in arguably the most recognisable phage, *Escherichia* phage T4 (Figure 1) [17]. One pentameric vertex is replaced by a dodecameric portal complex, enabling the packaging of the genome and providing an attachment point for the assembled phage tail. The linear DNA genome is packaged into the capsid to a pressure of up to 6 MPa through the portal [18]. Most virions require additional proteins to reinforce and seal the particle, such as scaffolding, cementing, and plug proteins, as well as adaptor proteins for connecting the different modules in the virion architecture at the tail–head interface and towards the tip of the tail [14]. Depending on the phage, these adaptors may connect the tail tube to a large complex called a baseplate, tail fibre(s), and the tail tip complex [19–22]. These structures are important for phage–host interactions including attachment and DNA injection into the host cell [23,24].

## Phage tails as cell puncturing devices

Phages are immotile in the environment. In a random encounter with a bacterial cell that can serve as host, the phage needs to enslave the gene expression programmes of the host cell to produce new phage progeny. The phage has three big issues to deal with in order to get its genome into the cytoplasm, establishing (i) a translocation channel in the outer membrane of a Gram-negative bacterium, (ii) a translocation channel through the cell wall and inner membrane of Gram-negative and Gram-positive bacteria, (iii) sufficient potential energy stored in the packaging of the genomic DNA to drive the passage of the phage genomic DNA through the tail tube and into the cytoplasm.

In the case of the prototypical *Escherichia* virus T4, the translocation channel through the outer membrane is created by the tail directly piercing the membrane lipids. Phage T4 has a tail that functions as a contractile nanomachine, with a rigid inner tail tube housed within a compressible outer sheath. When the phage is triggered to contract, the outer sheath compresses so that most of the baseplate structure moves and ∼40 nm of the inner tube is exposed [35–37]. Biophysical analysis of analogous contractile systems [38] and theoretical calculations are consistent with the assumption that the contractile force generated through the compression of the outer sleeve is sufficient to power the tail tube through the outer membrane [37]. The tail tip then forms a hole in the peptidoglycan layer before dissociating [39]. Although the tail tube has sufficient reach to cross the distance of the periplasm to the inner membrane (∼30 nm) [40], cryo-electron tomography reconstructions show that after contraction, the inner membrane bulges towards the tail by ∼16 nm [41]. It is likely that the tape measure protein helps form a DNA translocation conduit directly through the inner membrane into the cytoplasm [41]. The dimensions of the tail tube are conducive to DNA translocation: the polymerised tail tube protein (TTP) forms a straight tube with a 4 nm internal diameter [42], and multiple charged residues lining the inner surface of the rigid tail tube makes it highly electronegative [43,44].

Non-contractile tails do not rely on mechanical disruption of the bacterial membrane(s) and cell wall to inject their genome. *Podoviridae*-like viruses have only a minimalist tail but deploy a channel *in situ* upon attachment that acts as an ejectosome. The DNA is actively pulled out of the particle by the ejectosome itself and the bacterial RNA polymerase [32]. *Siphoviridae*-like tails have a diverse range of tail tip structures which are employed in the penetration of their host [19,45–48]. The long, flexible *Siphoviridae*-like tail tube appears unchanged before and after ejection in recent high-resolution structures [33,48–52], suggesting that other structural proteins, such as the tape measure protein, are involved in genome ejection.

## The pliable tail tube structure of *Siphoviridae*-like phages

The defining characteristic of *Siphoviridae*-like phages is the long non-contractile tubular tail (‘siphon’ is Greek for ‘tube’). Tail length is determined by the tape measure protein and varies significantly: λ phage has a 150 nm tail, whereas the P23-45 and P74-26 phages have tails exceeding 800 nm [53,54]. In contrast with contractile tails, the atomic details of the *Siphoviridae*-like tails have only been elucidated recently, because of technical difficulties associated with such intrinsically flexible objects. For phages T5 and λ, structures of monomeric TTPs determined by X-ray crystallography and nuclear magnetic resonance (NMR), respectively, allowed interpretation of medium-resolution cryo-EM reconstructions of the tail tubes [49,51,55,56]. This revealed a close similarity with the inner tail tube component of contractile tails both at the level of the TTP fold and its organisation into stacked rings. The TTPs are usually hexameric, but occasionally have only a pseudo-hexameric symmetry based on trimers formed from tandem duplications of the TTP domain, as seen in the T5 tail [49,57].

**Figure 2.**
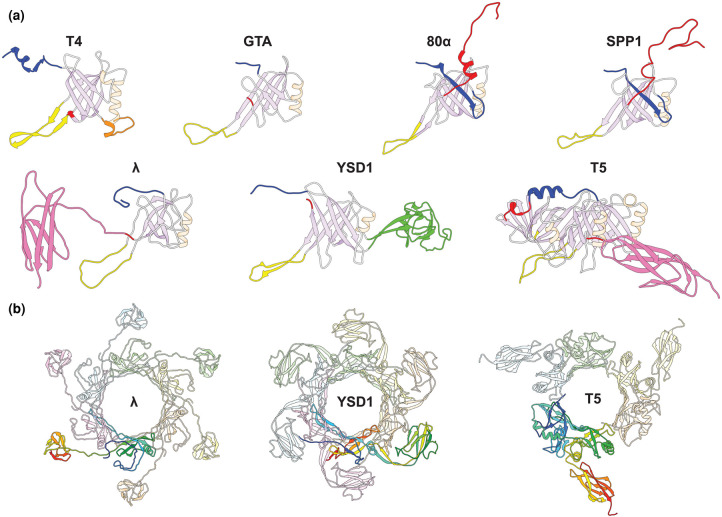
Structures of *Siphoviridae*-like TTPs. (**a**) Structures of TTP subunits from different *Siphoviridae*-like tails highlighting regions involved in quaternary interactions: N-terminus (blue), long-loop (yellow), extended helix/loop in T4 (orange), domain 2 in YSD1 (green), and C-terminus (red). Other α-helices and β-sheets are coloured in pale orange and lilac, respectively. The Ig-like domains of λ and T5 are highlighted in pink. (**b**) Hexameric rings of *Siphoviridae*-like TTP structures with a single subunit coloured in a rainbow representation from N- to C-terminus. Structures: T4 gp19 (PDB: 5W5F) [44]; *Rc*GTA g9 (PDB: 6TEA) [52]; 80α gp53 (PDB: 6V8I) [48]; SPP1 gp17.1 (PDB: 6YEG) [50]; λ gpV (PDB: 6P3E) [51]; YSD1 YSD1_22 (PDB: 6XGR) [33]; and T5 pb6 (PDB: 5NGJ) [49]. Images were generated in UCSF Chimera version 1.14 [34].

Each TTP subunit consists of a core β-sandwich domain, containing eight antiparallel β-strands (β1–β8), flanked by an α-helix and an extended hairpin loop between β2 and β3 (Figure 2a). The inner β-strands of the β-sandwich interconnect between the six subunits to form a continuous 24-stranded β-barrel. The N-terminus and C-terminus are the most variable components between viruses and are involved in intra- and inter-ring interactions, with the C-terminus sometimes incorporating additional, immunoglobulin (Ig)-like domains (Figure 2a,b).

The first reconstructions of *Siphoviridae*-like tail tubes that allowed *de novo* modelling of the assembled structure were obtained in 2020 for three phages which have relatively straight tail tubes: 80α, SPP1, and YSD1, as well as the phage-like *Rhodobacter capsulatus* gene transfer agent (*Rc*GTA) (Table 2) [33,48,50,52]. While there is great diversity of modules decorating the tip of the tail tube (e.g. baseplates, fibres, needles) depending on the specific phage, the tail tube itself is remarkably conserved, both at the level of the TTP fold, as well as its helical organisation. For instance, the helical rise in the various phages studied only varied between 38 and 43 Å, with twists of 17–24° (when considering T5 pb6 as a pseudo-hexamer), despite substantial phylogenetic distance. The conserved similarities extend also to other tube-forming apparatus that do not function as DNA conduits [52,55,58]. These other molecular machines, including killing devices such as R- and F-type pyocins and bacterial Type VI secretion systems, have been discussed in several excellent reviews [23,59,60] and this review will instead focus on aspects specific to the long and flexible tails recently elucidated.

**Table 2.** *Siphoviridae*-like TTP structures determined by cryo-EM

Phage	TTP	Method(s)	Helical rise/twist	Resolution	EMDB ID	PDB ID	Year	Reference
T5	pb6	cryo-EM/X-ray crystallography	40.6 Å/39.1° (C3)	6.2 Å (pre-ejection native tail) 5.8 Å (post-ejection native tail) 8.8 Å (TTP-only assembly)	EMD-3689 EMD-3690 EMD-3691	5NGJ	2017	[49]
SPP1	gp17.1	cryo-EM/NMR	38.5 Å/21.9° (C6)	4.0 Å (TTP-only assembly)	EMD-10792	6YEG	2020	[50]
λ	gpV	cryo-EM/NMR	42.8 Å/17.5° (C6)	5.4 Å (head-less tail assembly) 6.4 Å (pre-ejection native tail) 6.8 Å (post-ejection native tail)	EMD-20241 EMD-20242 EMD-20243	6P3E	2020	[51]
YSD1	YSD1_22	cryo-EM	41.2 Å/19.7° (C6)	3.5 Å (pre-ejection native tail)	EMD-22183	6XGR	2020	[33]
80α	gp53	cryo-EM	39.7 Å/21.3° (C6)	3.7 Å (head-less tail assembly)	EMD-20873	6V8I	2020	[48]
*Rc*GTA	g9	cryo-EM	38.3 Å/24.4° (C6)	3.9 Å (pre-ejection native tail) 3.8 Å (post-ejection native tail)	EMD-10478 EMD-10566	6TEA 6TSV	2020	[52]

## Peripheral tail tube domains

When comparing *Siphoviridae*-like TTPs, additional C-terminal domains or extensions often decorate the conserved structural core on the outside of the main tube. In phages SPP1 and 80α, an extended C-terminal arm projects from the main body of TTP to form inter-ring contacts as described in the next section [48,50]. In phages λ, SPP1, T5, and YSD1, an Ig-like domain projects outwards from the tail tube to give a rugged morphology to these tails (Figure 2b) [33,49,51]. The density for the C-terminal Ig-like domain in reconstructions of phages λ and YSD1 is weaker than the central tube, likely due to flexibility of this domain relative to the core domain, and averages poorly during helical reconstruction [33,51]. For YSD1, this domain was not modelled but the TTP contains an additional Ig-like domain inserted between strands 4 and 5 of the inner and outer sheets of the core domain, wrapping around adjacent subunits in an analogous position to the C-terminal Ig-like domains of phages λ and T5 (Figure 2b) [33].

Immunoglobulin-like (Ig-like) domains are found in five functional classes of structural proteins in phage virions: tail fibre proteins, baseplate proteins, TTPs, major capsid proteins, and highly immunogenic outer capsid (Hoc) proteins. From a curated dataset of phage genomes, Fraser et al. [61] identified 68 Ig-like domains encoded in the genomes of 41 tailed phages, representing a presence in ∼25% of the total number of genomes analysed. These Ig-like domains, whilst showing an overall conserved structural fold, are very different in sequence and are broadly classified into three superfamilies: bacterial Ig-like domain (Big_1, Big_2), immunoglobulin superfamily (I-set), and fibronectin type III (FN3) [61]. Given the significant mosaicism observed for proteins that carry these domains, certain functional classes of phage structural proteins only contain domains of a specific Ig-like family within their protein sequence. This is evident for tail fibre proteins and baseplate proteins, such as the receptor binding protein gpJ from phage λ that contains a FN3 domain [61], or the T4 phage Hoc protein that may contain several I-set domains [62]. *Siphoviridae*-like TTPs are the exception, as they may accommodate a single C-terminal Ig-like domain from any of the major superfamilies (Table 3). Whilst the majority of Ig-like domains are encoded in frame with the gene encoding the structural protein, many are incorporated into the protein by programmed ribosomal frameshifting, leading to a level of control over their incorporation into the virion structure [61]. For example, the *Bacillus subtilis* phage SPP1 carries two different TTPs: gp17.1 and gp17.1*, the latter the result of a +1 frameshift leading to the incorporation of an FN3 domain at the C-terminus of the protein [63].

**Table 3.** Examples of Ig-like domain containing major tail proteins

Ig-like domain	Phage	Protein name	Accession number	Bacterial host (genus)
Big_2	T5	pb6	YP_006973	*Escherichia*
	λ	gpV	P03733	*Escherichia*
Big_1	Chi	gp22	YP_009101117	*Salmonella*
	YSD1	YSD1_22	YP_009833591	*Salmonella*
I-set	HK97	gp12	NP_037706	*Escherichia*
	BP-4795	ORF64	Q6H9U0	*Escherichia*
	PY54	gp12	NP_892057	*Yersinia*
FN3	SPP1	gp17.1*	YP_710298	*Bacillus*
	Barnyard	gp30	NP_818568	*Mycobacterium*

Recent cluster analysis of sequences (CLANS) of putative TTPs from the 20 *Siphoviridae* clusters that infect *Enterobacteriaceae* [64] indicated that ∼70% of their TTPs contain Ig-like domains, including Big_1, Big_2, and I-set domains (Ig_1, Ig_2, and Ig_3) [33]. Despite their abundance, the specific function of these Ig-like domains for phage TTPs is unclear, but studies have shown that while these domains are not essential for tube formation or for infection of its host [63,65], some mutants lacking an Ig-like domain show a substantial reduction in infection efficiency compared with the wild type phage [56]. Given that these domains are exposed on the surface of the virion in high numbers, and because Ig-like folds are predominantly involved in intermolecular interactions, it has been speculated that they play an accessory role in recognising host surface molecules to aid adsorption [66] or, in the case of the T4 phage Hoc protein, binding to mucin glycan residues within the gut mucosal layers promoting persistence and subsequent infections of its bacterial prey within these environments [67].

## Robust but flexible tails

The genes encoding tail proteins are clustered together in the genome, and the gene order is conserved between different phages. This has led to the proposition that they form a genetic module that can be exchanged between viruses and their hosts [68,69]. In accordance with this mix-and-match hypothesis, *Siphoviridae*-like tails are known to be assembled independently and before attachment to the forming capsid [70]. Initially, chaperone proteins coat the length of the tape measure protein, and this template attaches to the tail tip or the baseplate [71,72]. Subsequently, TTPs displace the chaperones assembling sequentially in a helical stack of hexameric rings [71,72]. Finally, tail completion proteins form a ring to cap the tail tube and enable its attachment to the assembled capsid [28,73-75].

**Figure 3.**
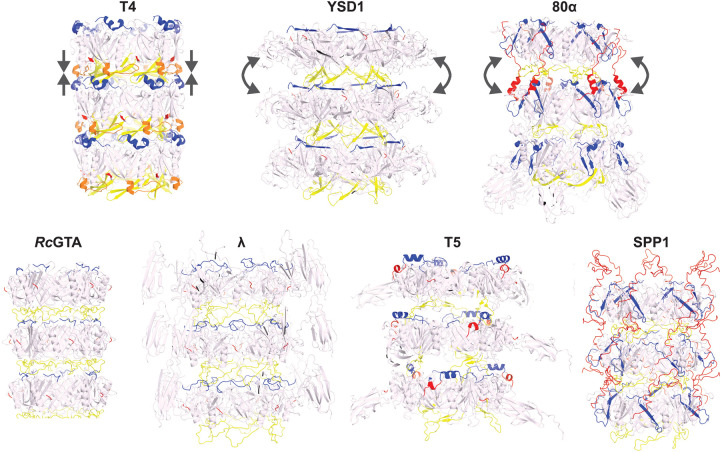
Structure of the tail tubes. Top row, representatives of three types of tail tubes: the inner tube of the compact contractile tails (T4), long flexible tails maintained primarily by the TTP long-loop (YSD1) or jointly by the long-loop and a C-terminal arm (80α). Bottom row: additional examples ranging between the two representative structures of *Siphoviridae*-like tails above. The core of TTP is coloured in pale pink, while protein elements located at the inter-ring interface are coloured in blue for the N-terminus, yellow for the long-loop and red for the C-terminal arm. The loop/helix in T4 that seals the inter-ring gap is shown in orange. Straight arrows indicate the compact packing in the smooth tail tube of T4; curved arrows represent the tilt observed in bent section of the tail tube. Structures: T4 gp19 (PDB: 5W5F) [44]; YSD1 YSD1_22 (PDB: 6XGR) [33]; 80α gp53 (PDB: 6V8I) [48]; *Rc*GTA g9 (PDB: 6TEA) [52]; λ gpV (PDB: 6P3E) [51]; T5 pb6 (PDB: 5NGJ) [49]; and SPP1 gp17.1 (PDB: 6YEG) [50]. Images were generated in PyMOL (The PyMOL Molecular Graphics System, version 2.5.0; Schrödinger, LLC).

The flexible tails found in *Siphoviridae*-like phages are characterised by prominent grooves between successive rings that depart from the smooth, continuous TTP layer of the T4 contractile tail (Figure 3). This morphological feature is the result of less extensive inter-ring interactions for the core TTP domain, mediated primarily by a loop projecting out from the β2 and β3 strands — or β3 and β4 for some viruses — in the main β-sandwich of TTP (called ‘long-’, ‘stacking-’, ‘extended-’, or ‘β-hairpin-’ loop depending on the phage). The long-loop interacts with the N-terminus of TTP of the next ring and mediates most of the contact so that in its absence, in most Siphoviridae-like tails, no stable assembly is predicted by protein interface analysis [33]. In phages 80α and SPP1, the rings are also interconnected through a C-terminal extension that forms a molecular arm reaching out to bind the outer side of the next ring [48,50] (Figure 3). The long-loop is also found in contractile tails (e.g. in phage T4), but it mediates more extended interactions involving an additional internal loop located immediately after the conserved α-helix of TTP [27,44]. The resulting stacking of the rings is compact, missing the characteristic inter-ring space of *Siphoviridae*-like tails.

A hybrid structure of phage SPP1 formed through combining solid-state NMR and analysis of variance in cryo-EM reconstructions, identified two hinge regions in TTP [50]. The long-loop forms the first hinge which is able to stretch to accommodate torsion in bent sections of the tail. Both phages SPP1 and 80α have an additional tether formed by a C-terminal arm in TTP that provides a second hinge between rings [48,50]. Distortions introduced during bending introduce an offset of ∼9 Å between the inner and outer sides of the rings, resulting in a average curvature radius of 655 Å. Mechanistically, the side of the ring on the inside of the bend is unchanged while the C-terminal arm extends on the outer side allowing a tilt to form [50].

Positioning of the long-loop and N-terminus on opposite sides of the ring is a pre-requisite for tail polymerisation. In isolation as monomeric proteins, TTPs are stable as judged in analysis by solution NMR [55], X-ray crystallography [49], and small-angle X-ray scattering (SAXS) [33]. Several TTPs self-assemble spontaneously in the absence of other proteins but at a slow rate [49,76]. It remains unknown what triggers the shift towards stable ring formation *in vitro* and *in vivo*. If a common molecular switch exists in the TTP, it is likely to be either the N-terminus or the long-loops, since these are the only two elements consistently differing between the assembled and in-solution forms of TTPs [33,49,51,55]. In most structures of monomeric TTPs the long-loop is disordered or flexible [33,49,51,55]. A plausible mechanism for nucleation proposes that a factor in the tail tip — such as the distal tail, terminal domain of the tape measure protein or the tail chaperones — permits the presentation of the N-terminus on the growing end of the tail.

In support of this hypothesis, mutants of the TTP from phage YSD1 suggest that, in the absence of other factors, the long-loop is a negative regulator of ring-like structure assembly, while the N-terminus is required for self-assembly [33]. On the other hand, once properly positioned, the long-loop is essential to axial polymerisation of the tail [49,76] and mutants of the λ and SPP1 long-loop are dominant negative [55,76].

## Interior surface of the tail facilitates genome ejection

Phages have been analysed structurally before and after DNA ejection. Low and medium resolution cryo-EM reconstructions had previously suggested differences in the TTP might be caused by DNA ejection from phages SPP1 and, to a lesser extent, λ [51,77]. However, higher resolution cryo-EM analysis of phage T5 and *Rc*GTA [49,52] showed no such conformational differences in the tail before and after ejection of the DNA. Overall, this suggests that the signal for DNA release is not transmitted through the TTP and strengthens an alternative hypothesis assigning this role to the tail tape measure protein [45,49,51]. Indeed, the tape measure protein is perfectly positioned in the virion to assume this role: it provides a physical link between components of the tail proximal to the head, and the distal end of the tail that carries host-interacting functions through the baseplate, tail tip, and/or fibre components.

Studies on the phage YSD1 tail tube structure suggests that physicochemical properties actively promote the transit of the ∼10 000 nm phage genome through the 220 nm long, 7 nm wide corridor [18,33]. If the tail tube is a passive conduit, a massively long DNA molecule should lose substantial velocity to the friction associated with those few hydration layers of water between the DNA and the inner surface of the tail tube [78]. Inamdar, Gelbart, and Phillips [78] formulated a general diffusion equation to describe pushing and pulling effects on the DNA based on reasonable assumptions made in the absence of detailed structural constraints. A further issue was raised by Sao-Jose et al. [79] considering the turgor pressure of the cytoplasm to realise that these calculations can only explain the entry of ∼15% of the genome length. However, uncertainties in the original calculations concerned the exact dimensions of the tail tube and the charge properties of the internal surface, which were at the time unknown.

**Figure 4.**
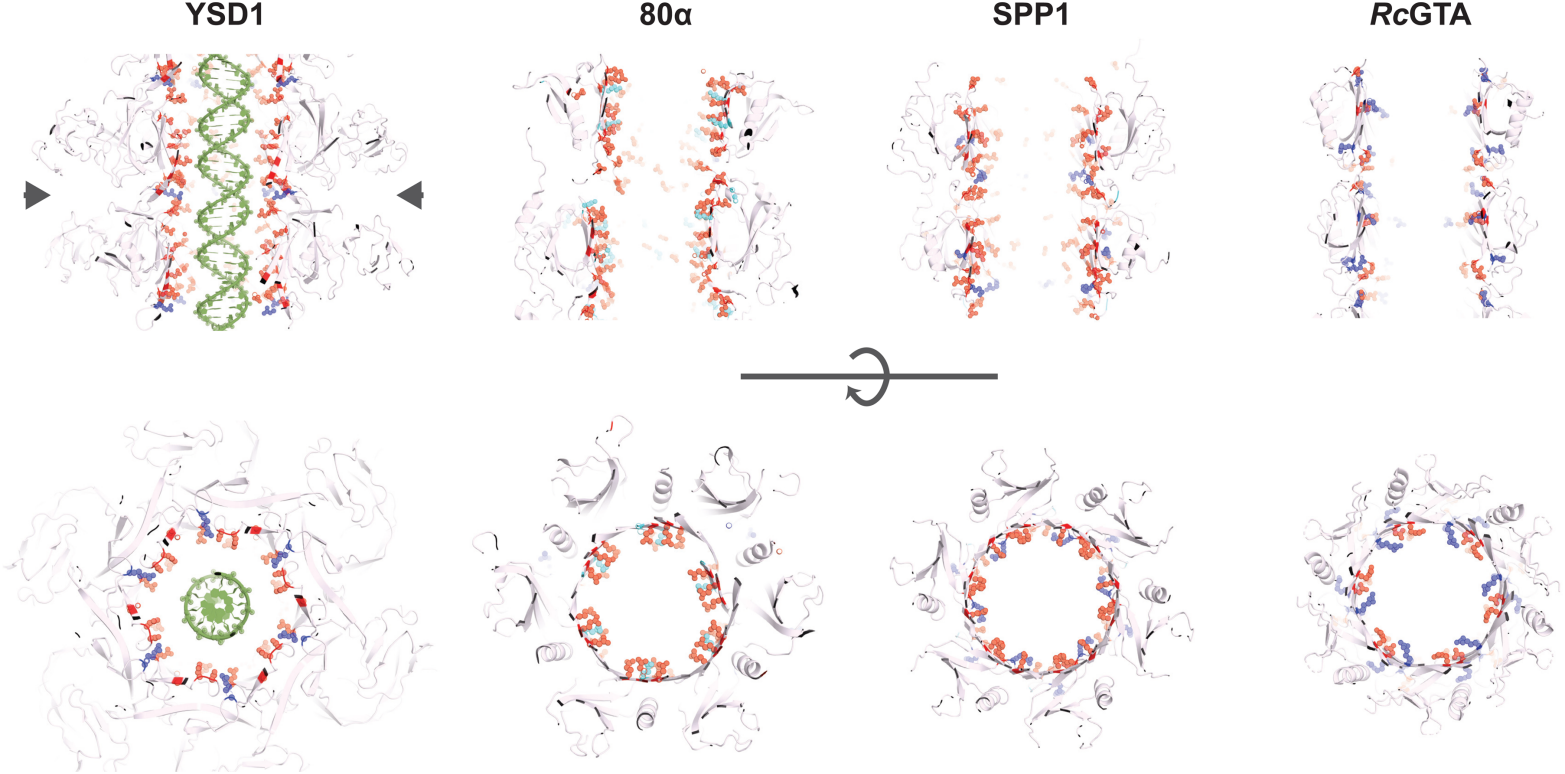
A pattern of acidic motifs decorate the interior of most tail tube involved in DNA ejection. Orthogonal views of four *Siphoviridae*-like tail tubes. The cross-sections shown in the bottom row approximately correspond to the region indicated by the arrow heads. The side chains of charged residues exposed onto the interior surface of the tail are shown as spheres. Glutamate and aspartate residues are coloured in red; lysine and arginine in cyan and navy blue, respectively. A generic double-stranded DNA molecule in its B-form has been modelled in the YSD1 tube. Structures: YSD1 YSD1_22 (PDB: 6XGR) [33]; 80α gp53 (PDB: 6V8I) [48]; SPP1 gp17.1 (PDB: 6YEG) [50]; and *Rc*GTA g9 (PDB: 6TEA) [52]. Images were generated in PyMOL (The PyMOL Molecular Graphics System, version 2.5.0; Schrödinger, LLC).

The inner surfaces of phage tail tubes are generally described as electronegative, which is hypothesised to minimise interactions with the DNA as it glides through the tube [45]. A detailed analysis of the tail tube structure of phage YSD1 at 3.5 Å resolution showed an extensive and highly patterned placement of acidic residues (Figure 4) [33]. It suggests that molecular features of the tail could contribute to potentiate the exit velocity of the genomic DNA once the initial impetus from the pressurised environment of the capsid begins to wane [33], and is consistent with a proposed theoretical requirement of DNA to ratchet from the tail tube using a specific arrangement of negative charges [78].

Tail tube interiors for phages YSD1, 80α, SPP1, and T5 present analogous patterns, observed to include dyads of aspartic/glutamic residues in close proximity, as well as one or two positively charged residues at the inter-ring connection (Figure 4) [33,48–50]. These patterns have no recognisable sequence conservation and might instead have been selected independently in the course of evolution due to similar mechanistic constraints.

## Conclusion

**Figure 5.**
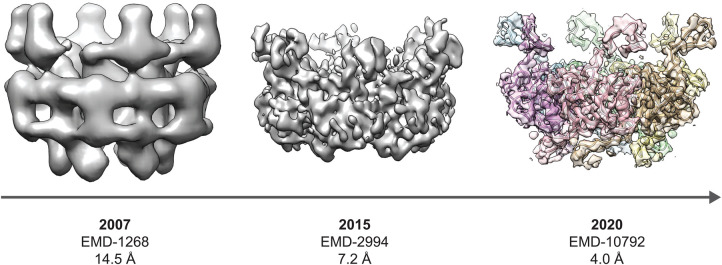
Progress in cryo-EM reconstructions of *Siphoviridae*-like phage tail tubes as exemplified by SPP1. EMD-1268 and EMD-2994 were cropped to density corresponding to a single ring using a mask generated from the structure of SPP1 gp17.1 (PDB: 6YEG) which was low-pass filtered to 20 Å [50,77,80]. EMD-10792 is coloured in a rainbow representation with each segment corresponding to a chain in the refined structure of gp17.1 [50]. Images were generated in UCSF Chimera version 1.14 [34].

The contractile tail of *Myoviridae*-like phages has been centre stage for decades, providing a paradigm for phage DNA injection that is both striking and amenable to robust structural analysis. Reminiscent of the reeds in Aesop's fable, *Siphoviridae*-like tails do not rely on brute force for stability and genome injection. They bend — but do not break — to provide a conduit purpose-built for DNA transit. How they achieve this is no less fascinating than their ‘more muscular’ (‘myos’ means muscle in Greek) counterparts in the *Myoviridae-like* phages. While it is not known whether the apparent flexibility has any functional consequences, one could speculate that once the phage is tethered to the bacterial surface by means such as interactive Ig-fold domains, flexibility in the tail might increase the capture-radius for the tail tip to encounter its receptor. A mechanistic understanding of these intrinsically flexible tails has required more sophisticated biophysical approaches, including advances in cryo-EM methodologies (Figure 5) and complementary techniques, such as solid-state NMR, SAXS, and X-ray crystallography.

## Perspectives

- *Importance of the field:* The TTPs of phages have evolved different strategies to transport their genomes across the cell membrane. Structural biology methods such as cryo-EM have been instrumental in determining the building blocks of these assemblies and the tail architecture.
- *Current state of the research:* Recent cryo-EM structures have revealed the structural motifs essential for *Siphoviridae*-like tail tube assembly; however, several questions remain: What is the molecular mechanism of nucleation of tail assembly? What is the structure of the tape measure protein in the assembled tail, and how does it interact with the TTP? How do host interactions with the tail trigger DNA ejection from the head through the tail?
- *Future directions:* To understand the mechanisms of tail assembly and genome release, the field is developing towards single-virion and time-resolved approaches. Current emphasis makes biophysical modelling, cryo-electron tomography, and solid-state NMR appear as methodologies of choice.

## Abbreviations

Cryo-EM: cryo-electron microscopy
EMDB: Electron Microscopy Data Bank
Ig-like: immunoglobulin-like
NMR: nuclear magnetic resonance
PDB: Protein Data Bank
*Rc*GTA: *Rhodobacter capsulatus* gene transfer agent
SAXS: small-angle X-ray scattering
TTP: tail tube protein
